# Multidimensional differentiation in foraging resource use during breeding of two sympatric top predators

**DOI:** 10.1038/srep35031

**Published:** 2016-10-11

**Authors:** Guilad Friedemann, Yossi Leshem, Lior Kerem, Boaz Shacham, Avi Bar-Massada, Krystaal M. McClain, Gil Bohrer, Ido Izhaki

**Affiliations:** 1 Department of Zoology, George S. Wise Faculty of Life Sciences, Tel Aviv University, Israel; 2 National Natural History Collections, The Hebrew University of Jerusalem, Israel; 3 Department of Biology and Environment, University of Haifa at Oranim, Kiryat Tivon, Israel; 4 Department of Civil, Environmental and Geodetic Engineering, The Ohio State University, Columbus, OH, USA; 5 Department of Evolutionary and Environmental Biology, Faculty of Natural Sciences, University of Haifa, Israel

## Abstract

Ecologically-similar species were found to develop specific strategies to partition their resources, leading to niche differentiation and divergence, in order to avoid interspecific competition. Our study determines multi-dimensional differentiation of two sympatric top-predators, long-legged buzzards (LLB) and short-toed eagles (STE), which recently became sympatric during their breeding season in the Judean Foothills, Israel. By combining information from comprehensive diet and movement analyses we found four dimensions of differentiation: (1) Geographic foraging area: LLB tended to forage relatively close to their nests (2.35 ± 0.62 km), while STE forage far from their nest (13.03 ± 2.20 km); (2) Foraging-habitat type: LLBs forage at low natural vegetation, avoiding cultivated fields, whereas STEs forage in cultivated fields, avoiding low natural vegetation; (3) Diurnal dynamics of foraging: LLBs are uniformly active during daytime, whereas STEs activity peaks in the early afternoon; and (4) Food-niche: while both species largely rely on reptiles (47.8% and 76.3% for LLB and STE, respectively), LLB had a more diverse diet and consumed significantly higher percentages of lizards, while STE consumed significantly higher percentages of snakes. Our results suggest that this multidimensional differentiation allows the spatial coexistence of these two dense populations in the study area.

Interspecific competition occurs when sympatric species consume or occupy a common limited resource essential for their survival or reproduction^1^. Interspecific competition has long been recognized as an important factor for shaping species distributions, and as an ecological force by which major modification in species communities can be shaped^2^. It can influence species abundance and distribution, habitat colonization rate, population size, species diversity, and even species extinction rate^2^,^3^,^4^. One strategy to avoid competition in sympatric species involves multidimensional niche differentiation^5^,^6^. Understanding the degree of niche overlap and the differences between coexisting species across multidimensional resources that include diet, space, and time is necessary to manage and preserve species’ populations in general and, as we show here, raptor populations in particular^7^.

Diet similarity between species, coupled with limited supply of resources, has long been recognized as one of the essential conditions of competition^8^. While dietary overlap indicates a potential for interspecific competition^6^, conclusive evidence for competition is typically based on manipulative experiments^9^, which are, in most cases, not feasible when studying top predators in the wild^10^. Competition theory predicts that diet similarity should be reduced, in neighboring pairs of different species, which breed in the same habitat^11^, compared to non-neighboring pairs. Body size and trophic structure are among the factors that determine the size of prey captured^12^ and therefore have a major impact on the competitive strength of sympatric species^13^. Yet ecologically-similar species have been found to partition their use of resources, such as diet, leading to niche divergence^14^.

In addition to having similar diets, spatial overlap of foraging habitats by species may also indicate potential for competition^15^. Spatial divergence between co-existing species’ foraging areas was observed as a mechanism to avoid competition for similar resources^16^,^17^,^18^. Foraging-habitat partitioning can be achieved by simple spatial differentiation at a landscape (geographic) scale, where each species utilizes different areas^18^,^19^. It can also be achieved at a small (patch) scale, where both species forage in the same geographic area but each forages in different sub-habitat (patch) type^20^. However, only a few studies have combined these two spatial dimensions when analyzing differentiation in foraging of coexisting species^16^,^21^.

Diel differences in activity time have been considered as the most important niche axis, apart from diet and habitat, along which organisms most frequently segregate^16^. Temporal partitioning may act either through exploitation competition, which presumes partitioning of other niche axes (mainly food and habitat), or through interference competition, which allows time to act as an independent niche dimension over which organisms may reduce the effects of agonistic interactions^22^. Thus, temporal segregation in foraging activity may be used as a mechanism of coexistence in sympatric species^16^,^22^.

Here, we present a study on the long-legged buzzard (*Buteo rufinus*) (hereafter LLB) and the short-toed eagle (*Circaetus gallicus*) (hereafter STE) - two diurnal, large-sized raptors occasionally sympatric throughout their breeding distribution^23^. In our study area, the Judean Foothills, Israel, these two species became sympatric during their breeding season recently, during the last two decades. It is assumed that this sudden change in community dynamics was a result of a LLBs’ population shift into this area^24^. Each year these two species return from their wintering areas to their sympatric breeding grounds in order to nest. Their populations have a large temporally overlapping breeding season, sharing the same areas for their nesting territories and, in some cases, even alternating use of nests in consecutive years^25^. Hence, it was questioned how LLBs which recently shifted into the STEs’ traditional nesting area coexist with each other? The goal of this study is therefore to determine the mechanism of coexistence for these two overlapping populations.

We investigate the possible mechanisms of resource partitioning that may explain the coexistence of these two sympatric top predators during their breeding season. Specifically, we aim to quantify the overlap between the two raptors along four dimensions: (a) large-scale foraging area, (b) small-scale foraging-habitat type, (c) diet, and (d) the diel timing of foraging. According to the niche-complementarity hypothesis, a high overlap in one dimension should be compensated by a low overlap in at least one other dimension^26^. We use this information to assess the likelihood of competition between the two species and to make recommendations for their management and conservation. We combine comprehensive diet analysis with GIS data analysis and high-frequency GPS animal-tracking data which provide millions of precise and reliable data points. Our study provides new insights into the spatial and temporal distributions and habitat preferences of coexisting animals^27^.

## Results

### Nest density and localization

The number of LLBs nests in the breeding area increased by 38% from 2007 to 2014, whereas the number of STE nests remained relatively stable (62 ± 1.5, mean ± SE) during the four study seasons (2011–2014, Fig. 1). We analyzed the average distance to the nearest neighboring nest, based on the 2013 breeding season. We found that the interspecific average distance between LLB to the nearest STE nest (1.44 ± 0.27 km, n = 37) and STE to the nearest LLB nest (1.30 ± 0.18 km, n = 63) were relatively similar. However, the intraspecific distance to nearest neighbor among LLB nests was significantly larger than that of STE nests (2.65 ± 0.29 km, n = 37 and 1.23 ± 0.16 km, n = 63, respectively, t = 4.2, P < 0.0001).

### Large-scale foraging area

**Figure 2:**
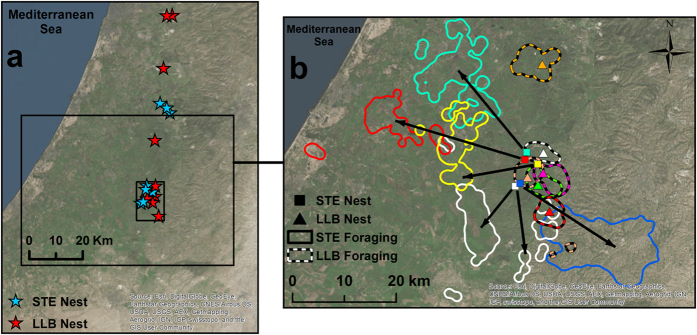
(**a**) Nest location of all tagged raptors (LLB n = 9, STE n = 8) at the entire study area. (**b**) Foraging areas (defined by a 95% KDE with a constant smoothing factor of 500 m) of 5 STEs and 6 LLBs. Note that 10 of the nests (marked inside the small rectangle in a) were located close to each other and that LLBs tended to forage close to their nests (2.35 ± 0.62 km), while STEs used “flight corridors”, represented by black arrows, to access their far foraging areas located at a mean distance of 13.03 km (±2.20) from their nests and at different geographical areas. These figures were generated by using GIS software - ArcGIS (ESRI. 10.1).

**Figure 3:**
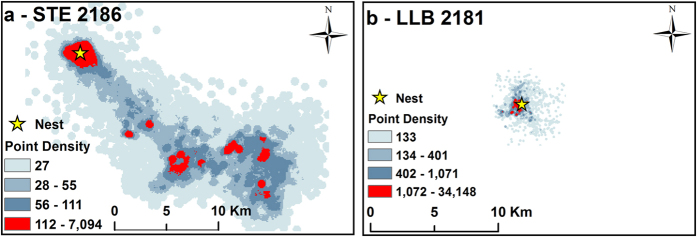
Point density analysis (smoothing factor of 500 m) of all movement data from one STE (#2186 a) and one LLB (#2181 b) during the entire breeding season. Note that the total area of STE’s movements was greater than LLB’s. In addition, STEs use “flight corridors”, of ~5–30 km, from their nests to their main foraging areas (**a**), while LLBs tended to forage close to their nests (**b**). These figures were generated by using GIS software - ArcGIS (ESRI. 10.1).

All reported GPS locations for both species were within a 116 by 78 km area, within the semi-arid and Mediterranean ecosystems of the Northern Negev, Judean Foothills, Coastal Plains and Western Judean Mountains in Israel (Fig. 2a,b). The average distance between the foraging areas of STE and its nest during the whole breeding season was 13.03 km (±2.20), and was 5.5 times greater than that of LLB (Table 1, Figs 2b and 3). Therefore, while LLBs intensively wander relatively close to their nests, STEs typically forage relatively far away from their nests (note the red areas in Fig. 3a,b).

Consequently, the average foraging area of STEs during the whole breeding season was 3.5 to 4.2 times larger than that of LLBs, and depended on the value of the Kernel Density Estimator (KDE^28^) smoothing factor (Table 1, Figs 2b and 3). The average foraging area of STEs during the shared breeding period was 2.5 to 3.0 times larger than that of LLBs, depending on the smoothing factor (Table 1, Figs 2b and 3). The average Utilization Distribution Overlap Index (UDOI^29^) during foraging among STE individuals (0.16 ± 0.19) was significantly higher than among LLB individuals (0.01 ± 0.01). It was also larger than the interspecific average UDOI between individuals of the two species (0.03 ± 0.07, F _2.26_ = 5.62, P = 0.01 followed by Bonferroni multiple comparison test, P < 0.05 on arcsin-sqrt transformed UDOI values).

**Figure 4:**
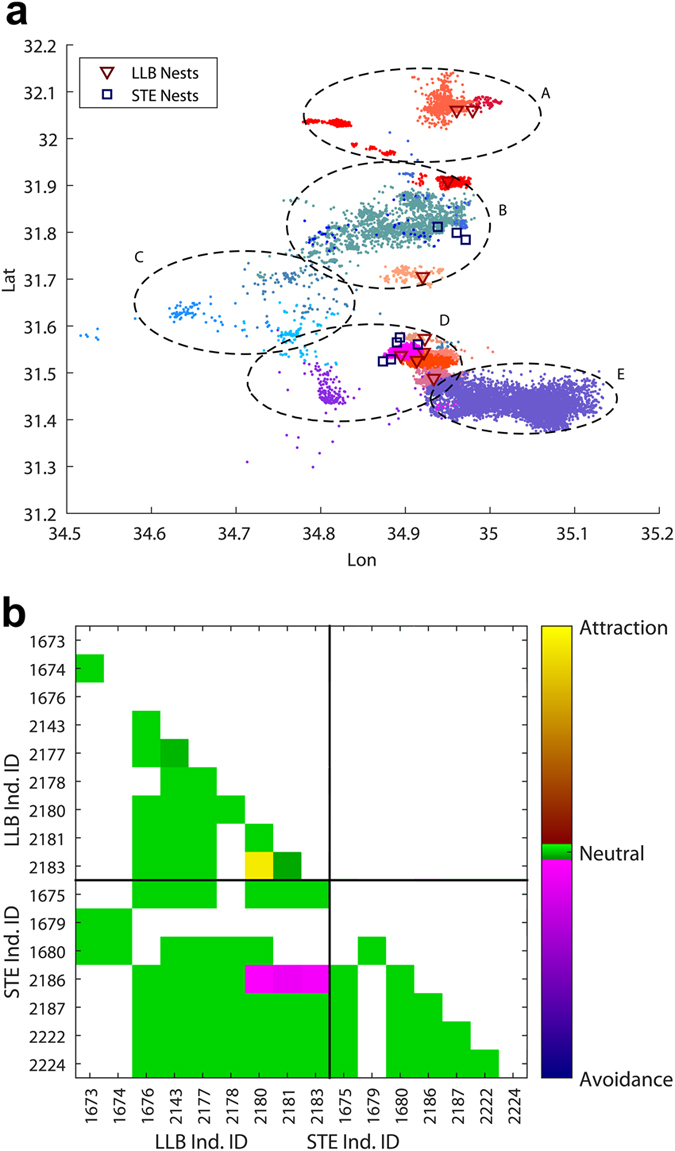
(**a**) Clustering of individual birds’ foraging locations. We have identified 5 significant foraging-locations clusters (dashed ellipses, labeled A–E). The foraging locations of each individual are marked by colored dots, with LLB individuals in “warm” shades (magenta-red-brown) and STE individuals in “cool” shades (violet-blue-grey). Nest location for each individual are marked by a triangle (LLB) or a square (STE). Cluster analysis to group foraging locations was conducted using the k-means function in MATLAB. (**b**) Results of attraction-repulsion analysis of movement patterns between each pair of individual birds using MoveMine^49^. Individual bird IDs are listed and sorted by species. The figure is symmetric across the diagonal. Color marks the attraction index, varying from 0 (complete avoidance) through 0.5 (mutually random movement pattern) to 1 (complete attraction, coordinated, matching movement patterns). A white (blank) square indicates that these two individuals were never observed at the same year. Most individuals display neutral movement patters to each other, except three LLB individuals that display avoidance toward STE individual 2186, and one pair of LLBs (2183 and 2180) that show matching (attracted) movement patters. This figure was plotted using MATLAB (Mathworks, Natick, MA) version R2015b (http://www.mathworks.com/products/matlab/).

A spatial cluster analysis on foraging locations showed that individuals’ foraging locations clustered by species (Fig. 4a, Supplementary Table S1). We identified five foraging clusters, two of which were nearly exclusively LLB (clusters A and D, Fig. 4a, Supplementary Table S1), another two which were exclusively STE (clusters C and E), and the fifth (cluster B) which was 75% STE.

Analysis of attraction/repulsion patterns in the movement data showed that most individuals where neutral to each other (Fig. 4b), indicating that direct intra- and interspecific interactions between individuals are rare. The exception to this general observation of no direct interactions were one STE’s (2186) movements that displayed slight repulsion toward three LLBs, and the movements of two LLBs (2180, 2183) that were highly synchronized and indicated attraction.

### Small-scale foraging habitat type

**Figure 5:**
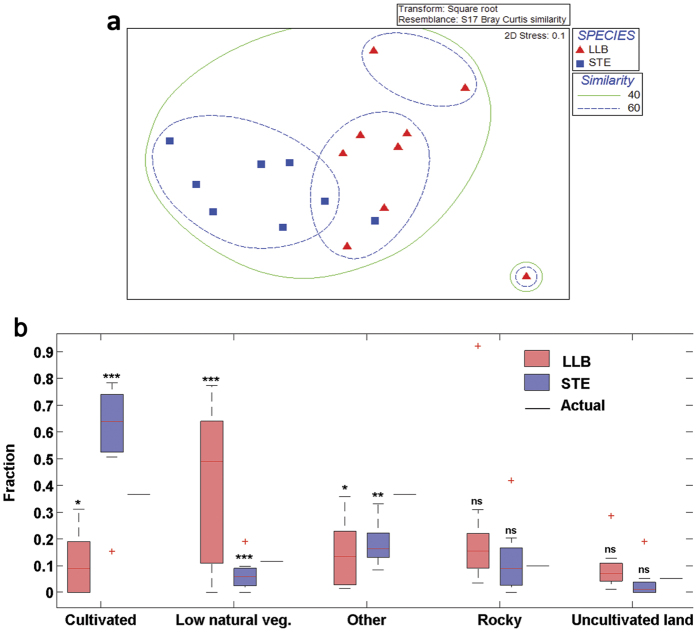
(**a**) Multi-dimensional scaling (MDS) plot of similarity in habitat type used for foraging by the two raptor species during the breeding season. Each point represents one individual. The habitat types of LLB and STE were significantly different from each other. The lines represent 40% and 60% similarity contours, so all samples inside each contour have a similarity above these two values, (**b**) Box plot of habitat type used by each species in comparison (one sample *t*–test) to habitat type availability in the whole study area (“Actual” line). Box = 25th and 75th fractions; bars = min and max values; red lines = median; red dots = outliers. Note that each species foraged in different habitat types; LLB showed a clear significant preference to low natural vegetation, and significantly avoided cultivated fields, while STE showed an opposite behavior by significant preference of cultivated fields and significant avoidance of low natural vegetation. *= 0.01 < P < 0.05, **= 0.001 < P < 0.01, ***= P < 0.001, ns = not significant (P > 0.05).

Ordination of the habitat-type dataset indicated a pronounced and significant difference (stress value = 0.1) in the overall composition of the types of habitat used for foraging, with low overlap between species (Fig. 5a). LLB over-utilized Mediterranean Garrigue and Batha (hereafter low natural vegetation), with this habitat comprising 41% of the full range of habitats used for foraging, even though it covered less than 12% of the study area. In contrast, LLB under-utilized cultivated fields (12% use versus 37% availability, Fig. 5b). STE exhibited the opposite pattern, over-utilizing cultivated fields (59% use versus 37% availability) and under-utilizing low natural vegetation (7% use versus 12% availability, Fig. 5b).

Multi-dimensional scaling (MDS) plots of similarity in habitat type showed that there is minimal overlap in the habitat-type preferences of the two species, with only a single STE falling within 60% similarity cluster of LLB (Fig. 5a). ANOSIM analysis confirmed the significance of the low degree of habitat type overlap between the two raptor species (Overall R = 0.44, P < 0.001). The average Bray-Curtis diet dissimilarity between the two raptor species was 49.7% (Supplementary Table S2). SIMPER analyses confirmed that most of this difference was due to the opposite preferences of cultivated fields by STE and of low natural vegetation by LLB (24.3% and 19.2% contributions for dissimilarity, respectively). Individuals of STE were more similar to each other (average 63.9%) than those of LLB (average 59.7%). Cultivated fields were the most typical foraging habitat of STE, contributing 50.1% to the average resemblance within the species whereas low natural vegetation was the most typical foraging habitat of LLB (30.3%, Supplementary Table S2).

The observed low overlap in foraging-habitat types between the two raptor species (O_jk_ = 0.24) was not significantly different than expected by chance under the null simulated values of randomization algorithms RA3 (Supplementary Table S3). The observed overlap of foraging habitats between individuals within each raptor species (O_jk_ = 0.39 for LLB and 0.38 for STE) was significantly higher than expected by chance by RA3 (Supplementary Table S3).

### Timing of foraging

**Figure 6:**
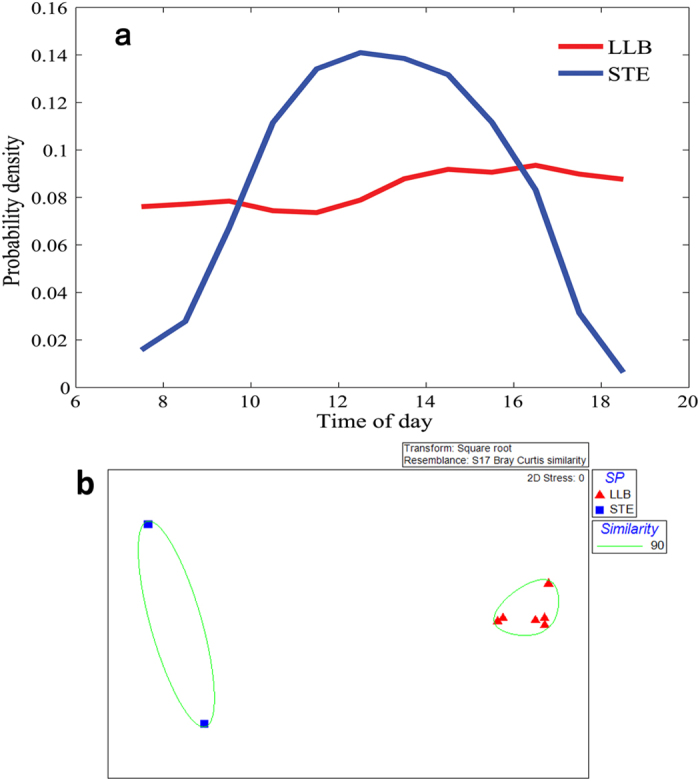
(**a**) Daily foraging time activity during 07:00–19:00 of each raptor species. Note that LLB tended to forage during all day time while STE showed a unimodal pattern with a clear midday peak. (**b**) Multi-dimensional scaling (MDS) plot of similarity in foraging time used by the two raptor species during the breeding season. Each point represents one individual. Although the patterns of the foraging time of both species largely overlap, these two species had different patterns of daily foraging activity.

Although the patterns of foraging time in both diurnal species were largely overlapping, the two species had significantly different patterns of daily foraging activity. LLBs demonstrated an almost uniform pattern of activity during daytime (between 07:00 and 19:00), while STEs exhibited a unimodal pattern with a clear midday peak (between 11:00 and 15:00) (Fig. 6a). MDS ordination clearly (stress value = 0) separated the foraging activity pattern of the two species (Fig. 6b) and overall R analysis confirmed significant differences between the two species (Overall R = 1.0, P < 0.05). SIMPER analyses demonstrated that the overall dissimilarity of foraging time between the two species is less than 18%, and most of this difference was due to the high and low activity of LLB and STE, respectively, in the early morning (between 07:00 and 08:00) and late afternoon (between 18:00 and 19:00, Supplementary Table S4). Both species demonstrated high intraspecific similarity in their foraging activity pattern with an average of 95.9% and 90.2% similarity in LLB and STE, respectively (Supplementary Table S4).

The high observed Pianka’s overlap index in foraging time between the two raptor species (O_jk_ = 0.86) was similar to that expected by random chance, as simulated by the RA3 algorithm (Supplementary Table S3). The high observed foraging-time overlap values between individuals within each raptor species (O_jk_ = 0.98 for LLB and 0.96 for STE) were significantly higher than expected by random chance (using RA3 algorithm, Supplementary Table S3).

### Diet

**Figure 7:**
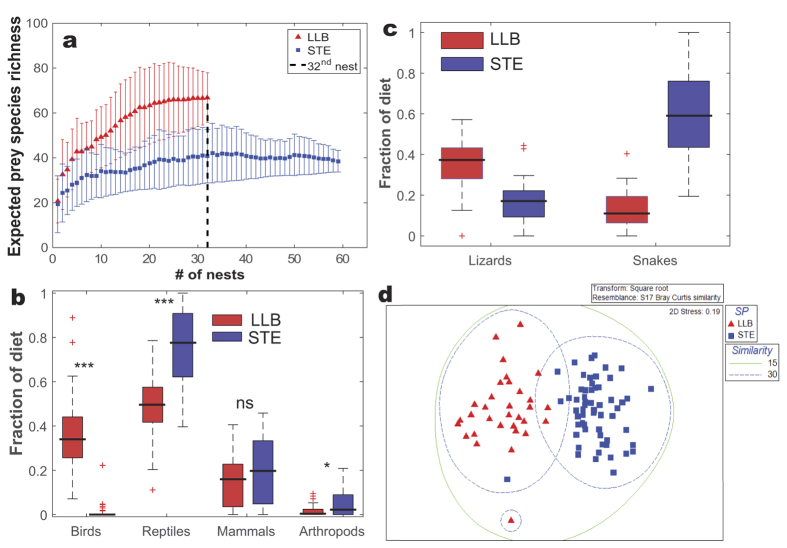
(**a**) Expected species accumulation curves based on Chao1 estimate of prey taxa richness of LLB and STE. Average and bootstrapped SD are based on 1,000 permutations. The curves appear to be approaching asymptote, indicating that most prey taxa were sampled. The vertical line indicates that when taking into account a similar sample size (n = 32 nests) more prey taxa were found in LLB than in STE diets. (**b**) Comparison (Independent sample *t*-tests) of the total fraction of each prey category (reptiles, birds, mammals and arthropods) between the two raptor species. *= 0.01 < P < 0.05, ***= P < 0.001, ns = not significant (P > 0.05). Box = 25th and 75th fractions; bars = min and max values; black lines = median; red dots = outliers. (**c**) Comparison (Repeated measures ANOVA) between the consumption of lizards (Sauria) *versus* snakes (Ophidia) between the two raptor species. Box = 25th and 75th fractions; bars = min and max values; black lines = median; red dots = outliers. Note that LLB consumed higher percentages of lizards, while STE consumed much higher percentages of snakes. (**d**) Multi-dimensional scaling (MDS) plot of similarity in dietary prey taxa of the two raptor species during the breeding season. Each point represents one nest. The diets of LLB and STE were significantly different, indicating that each species selected different proportions of prey items. The lines represent a 15% and 30% similarity contours, so all samples inside each contour have a similarity above these two values.

Based only on nests with a minimum of eight prey items, we identified a total of 1,416 and 1,239 prey items from 59 STE and 32 LLB nests, respectively, during three breeding seasons (2011–2013). The actual/observed prey-taxa richness found in LLB nests was higher than that of STE (49 and 34, respectively, Supplementary Table S5). Because the observed prey-taxa richness depends on sample size, we calculated Chao1 non-parametric estimators for the predicted prey-taxa richness. The curves for both species appear to be approaching the asymptote, indicating that the most common prey taxa were sampled. The rarefaction plots show that the Chao1-predicted prey-taxa richness of LLB is roughly 60% higher than that of STE (65.1 and 40.7, respectively, Fig. 7a) when taking into account the lower sample size (32 nests of LLB, see the vertical line in Fig. 7a). Dietary niche breadth of LLBs was 31% higher than that of STEs (*Bi* = 10.7 and 8.2, respectively).

Although the two raptor species share 30 prey items (Supplementary Table S5) the proportions of prey categories in their diet differ significantly, as indicated by the higher fraction of birds in LLB diets and the higher fraction of reptiles and arthropods in STE diets (Fig. 7b). No significant difference was found with regard to the fraction of mammals in the diets of the two species (Fig. 7b). Further analysis focusing on the composition of reptiles in the diet showed that LLB consumed a higher percentage of lizards (Sauria), while STE consumed a higher percentage of snakes (Ophidia) (Repeated Measures ANOVA, F_1,1_ = 161.3, P < 0.001, Fig. 7c).

Ordination of the dietary data set indicated a pronounced and significant difference (stress value = 0.19) in the overall composition of the diets of the two species with low overlap (Fig. 7d). Nonparametric MDS indicated that only two LLB nests fall within 30% similarity cluster of STE and three STE nests fall within 30% similarity cluster of LLB (Fig. 7d). ANOSIM confirmed the significantly low degree of dietary overlap between the two raptor species (Overall R = 0.77, P < 0.001). The average Bray-Curtis diet dissimilarity between the two species was high (71%, Supplementary Table S6). SIMPER analyses showed that two snake taxa (large whip snake, *Dolichophis jugularis*, and an unidentified large Colubridae) contributed most to this difference (15.7%), followed by pigeons (*Columba livia domestica*) and other unidentified birds (14.9%) and schneiders skink (*Eumeces schneideri*, 7.0%). Other prey types contributed 2–6% each to dietary dissimilarity. Individuals of STE were slightly more similar to each other (average 52%) than those of LLB (average 48%). Large whip snake was the most common prey item of STE, contributing 30.4% to the average resemblance within the species whereas roughtail rock agama (*Stellagama stellio*) and schneiders skink were the most common prey items of LLB (20.2% and 18.0%, respectively, Supplementary Table S6).

The observed medium dietary-niche overlap between the two raptor species was significantly higher than expected by random chance during two of the three years and for all years combined (RA3 simulation, *O*_*jk*_ = 0.48 Supplementary Table S3). The observed diet overlap between individuals within each raptor species (*O*_*jk*_ = 0.85–0.94 for LLB and 0.91–0.94 for STE) were significantly higher than expected by random chance for both species (RA3 simulation, Supplementary Table S3).

## Discussion

We present data suggesting a multi-dimensional niche partitioning between two raptor species which recently became sympatric within a small area of 215 km^2^ in the Judean Foothills during their breeding season. The nest density of STE, the veteran inhabitant of the area, is higher than reported in other areas in the world^30^,^31^ and is probably the highest in their entire breeding distribution. The nesting density of the LLB, which first arrived in this area twenty years ago, is probably the highest in their entire breeding distribution as well^32^,^33^,^34^,^35^. In some parts of our study area we recorded only 100–200 m between the nests of the two species. Furthermore, it appears that nest densities of both species are still increasing.

Because both species are top predators, both are active at daytime, and are similar in body type and activity patterns, it is expected that their new geographical overlap in the Judean area will create also an overlap over food, foraging areas, foraging habitats and foraging time. Furthermore, the close proximity of nests of the two species is particularly peculiar considering that LLB is known as an aggressive and territorial species, which is sensitive to interspecific and intraspecific intruders^36^. Here we demonstrated that the two species ecologically segregate across four niche dimensions: foraging area, foraging habitats, foraging timing and diet (Table 2).

GPS tags which operated more than two years showed high fidelity of both species to their nesting territories and foraging areas. Despite their high breeding densities and sympatric nesting sites, the GPS tags revealed a clear spatial pattern of interspecific segregation between the foraging areas of the two species. LLBs tended to forage relatively close to their nests. STEs intensively foraged relatively far from their nest, and used larger foraging areas than LLBs. Both species maintained low overlap among individuals’ foraging areas. Each species thereby segregates intra-specifically as well. Analysis of patterns of attraction-repulsion movements revealed that most individuals, regardless of species, were neutral to each other. Both intra- and interspecific interactions among individuals were thus rare.

STEs tended to use long “flight corridors” to reach their foraging areas. These corridors had never before been documented in this species’ breeding movement ecology. The furthest STE foraging point was sampled at a distance of 35.63 km from its nest.

Our results show that these two species spatially segregate at two distinct scales (Table 2). They choose geographically distinct foraging areas, and within each foraging area, each species targets a different land-cover type (habitat-type). Land cover is considered an important component in determining raptors’ foraging preferences^20^,^24^,^37^. LLB mainly foraged in low natural vegetation habitats, whereas STE mainly foraged in cultivated fields. However, in areas without LLBs, STEs are assumed to forage in low natural vegetation habitats^38^ and therefore, one possible explanation for the habitat segregation in our study area can be derived from a STE’s strategy to avoid competition in their sympatric foraging areas. On the other hand it can also be derived from interspecific differences in their visual attributes. Visual information is necessary to detect prey items against the background and track them visually until capture^39^. Thus, vision-related differences between the two species such as visual field configuration, the degree of eye movement, the size of the binocular and blind areas should affect the two species’ prey-searching strategies in each habitat type^39^, and thus affect their prey specialization (see below).

We found that the low observed overlap in foraging habitats between the two raptor species (*O*_*jk*_ = 0.24) was lower (but not significant) than expected by chance under the null simulated values of RA3 algorithm. This may indicate that the types of habitats, rather than the number of habitat types, reduced the ecological similarity between the two species. On the other hand, as expected, both species showed a higher intraspecific similarity than expected by chance in their habitat type preferences, which were also higher than the value of their interspecific similarity. This pattern of non-random foraging in available habitat types exhibited by the two species may emerge from interspecific competition, but may also be a natural consequence of other differences between the species, such as morphological differentiation and prey specialization.

Differences in timing of foraging may also be an important mechanism reducing interspecific competition among avian species^16^,^22^,^40^. There was a large overlap in foraging hours between the two species, and within each species, as both are diurnal raptors. However, foraging patterns clearly differed between these two species as indicated by the highly significant overall R and by the MDS ordination. LLBs were equally active during each hour of daytime whereas STEs showed a rather unimodal pattern.

It is unknown whether this temporal segregation is a direct consequence of a mechanism to reduce interspecific interaction^22^, an outcome of the different environmental conditions required by each species, or an outcome of the differences in diet and temporal availability of prey throughout the day. For example, high wing-loading species^41^ such as STEs may wait for thermal currents to develop in early mornings in order to fly to their foraging areas. On the other hand, better soaring conditions are typically available during midday and early afternoon, especially for high wing-loading species^41^. Therefore, species with lower wing-loadings, such as LLBs, are able to exploit weaker thermals and are expected to expand their activity time in the early morning and late afternoon. However, the temporal segregation between the two species may reflect the temporal activity pattern of their main prey items. For example, reptiles, the main prey of STE, are mainly active at midday, while birds, which are the main prey of LLB, are active throughout the entire day. Regardless of its direct cause, this timing segregation is clearly an important factor that influences the potential interactions between these two species.

As expected, the observed diet similarity between the two species was much lower than the observed diet niche overlap within each species. All these values were significantly higher than expected by chance and may indicate that the type, rather than the number, of dietary items increased dietary niche overlap not only within species, but also between the two species. However, these results may reflect the fact that the RA3 analysis assumes that all dietary categories were equally available to both consumers.

A possible explanation for the higher observed dietary niche overlap within STEs than within LLBs is that STEs display a low degree of reversed sexual dimorphism, which may be linked to its more specialized diet^3^, while LLBs display much higher degrees of reversed sexual dimorphism, were females are much larger than males, and thus their diet is more diverse within the population.

We found that LLB is more opportunistic than STE, as indicated by its wider dietary niche breadth and higher observed and predicted prey-taxa richness. The overall diet analysis showed a significant difference in the proportions of prey categories in the two species’ diets, as indicated by a significantly higher percentage of birds in the LLB diet and much higher percentage of reptiles and arthropods in STE diets. However, although the diet of both species largely rely on reptiles, we found that LLBs consumed significantly higher percentages of lizards, while STEs consumed significantly higher percentages of snakes. Hence, there is a clear segregation not only among prey categories but also in the composition of their reptile diet.

It is unclear whether this diet segregation derives from a niche shift process, which responds to interspecific competition, or from the morphological differences and hunting techniques used by these two species. For example, LLB is known to be an aggressive and rapid raptor, which dives to attack its prey diagonally^42^ and catches it by surprise, and thus can prey on flying birds and quick lizards^36^. STE is known to be a slower and cumbersome raptor, which dives to its prey vertically, sometimes with several aerial midair stopovers.

We propose that the diverse and rich diet of LLBs may facilitate its successful colonization in new breeding areas, as is the case in recent years in the Judean Foothills^24^. Such diet plasticity influences the ability to occupy new habitats^43^, as was documented in other raptors^10^,^44^ in which their plasticity in behavior and life history facilitates their expansion to new areas.

Although, STE is known as a specialist predator of snakes^30^,^38^,^45^, our results suggest that the diet of STEs in our study area is more diverse and expansive than it is in other breeding areas, with snakes comprising only 54.5% of its total diet. This may fit the predictions of competition theory^11^, and can lead to the assumption that in their former allopatric stage their diet similarity was higher but prey diversity was lower and similar to other areas where STE is a specialist predator. However, in their current sympatric stage, their diet similarity is reduced and their dietary-niche diversity is expanded. Thus, recent LLB population shifts and their increasing population density may have led to a decrease in STE prey availability, forcing them to add alternative prey to their diet.

The n-dimensional niche theory predicts that even minor segregations along several different dimensions can lead to a significant overall segregation^40^. Indeed, our study shows that the two raptor species significantly segregate on each of the four dimensions analyzed (Table 2). Despite nesting in close proximity, the two species demonstrated a clear spatial segregation in their foraging areas. Furthermore, multiplying the Bray-Curtis similarity values of the other three axes of differentiation (0.50 habitat type X 0.83 foraging active hours X 0.29 diet = 0.12), shows a clear niche segregation between these two species. Therefore, it seems that the two species could coexist in the same area.

Our results suggest a combination of four dimensions of segregation between two sympatric populations with similar ecology that had been allopatric during their entire history in the Judean area. However, it is impossible to rule out an alternative hypothesis - that pre-existing dissimilarity in these top predator niches, due to physiological differences, ghost of competition past, or any other long-term evolutionary driver, is the cause rather than the effect of the successful sympatric coexistence. Nonetheless, this study provides important insights on the ecological mechanisms which allow coexistence of two dense populations of top predators.

## Methods

### Study area and species

The study was conducted during three breeding seasons (2011–2013). The total study area (9,048 km^2^ of foraging area and 215 km^2^ of breeding area) comprised the Judean Foothills, the Western Judean Mountains, the Coastal Plain and Northern Negev, while the breeding area comprised several parts of the Judea region, Israel. The Judea mountainous region rises up to 800 m above sea level and is dissected by several streams flowing west to the Mediterranean Sea or east to the Dead Sea. On the west, the mountains border the Coastal Plain or Judean Foothills - a region of undulating low hills ranging in height between 150–450 m above sea level, where the natural plant community is comprised of Mediterranean Garrigue and Batha formations (hereafter “low natural vegetation”), as well as scattered trees (*Quercus calliprinos, Pistacia lentiscus* and *Ceratonia siliqua*). The valleys between these hills are mostly cultivated. For a full description of the two studied species, see Supplementary Methods.

### Nest localizations and nest visits

During the three breeding seasons we systematically located active nests of LLBs’ and STEs’ by following the breeding pairs and by systematically searching and inspecting all suitable nesting trees and cliffs within the study area. The majority of pairs did not nest in the same nest each year^25^, thus we needed to locate their new nests every year. By doing so, we have found 100 LLBs’ nests and 182 STEs’ nests total. We calculated the mean distance to the nearest neighbor between LLBs’ and STEs’ nests (interspecific) and within each species (intraspecific), by using the ESRI ArcGIS 10.1 ‘Near’-function. We analyzed the average distance between nearest neighboring nests for 2013 breeding season only, because this was the year with the largest number of observed STE nests (n = 63) and LLB nests (n = 37). Each one of the 282 active nests was visited and monitored at least twice throughout each breeding season: during the chick-rearing period (~30 days after hatching) and again two weeks after fledging.

### GPS tagging and tracking

Between 2011 and 2013, we trapped and tagged with GPS tags (E-Obs GmbH; Munich, Germany) 13 breeding LLBs (five regular and eight solar powered) and 11 breeding STEs (eight regular and three solar powered). The two types of GPS tags were assigned at random to each individual, regardless of species. Tags were fitted at the center of their back using special Teflon harness, and were programmed to sample one GPS fix (data point) at five minute intervals (for the solar tags) and 90 minute intervals (for the regular tags). Each fix provided date, hour, longitude, latitude, elevation, ground speed and heading azimuth. In addition, each tag included a specific UHF-signal (pinger) to facilitate tagged individual detection. These tags accumulated and stored GPS data until the data was downloaded through UHF communication. After the data were downloaded successfully, the tag continued to store new data until the next download. LLBs and STEs are diurnal raptors and thus these tags were programmed to work in a 12 h duty cycle (06:55–19:05) with 147 fixes per day (11 solar GPS tags, track duration: ~730 days: 2,697-39,592 fixes per individual for two breeding seasons: many of these fixes occurred outside foraging hours) or 4–10 fixes each day, mostly during foraging hours around midday (13 regular GPS tags, track duration: 75–450 days: 244–1,081 fixes per individual for one breeding season) due to battery and storage limitations. Therefore, both types of GPS tags provided comparable sample sizes for the analysis of foraging area.

The actual number of tagged individuals included for further analyses was eight STEs and nine LLBs, as we excluded individuals that did not achieve the chick-rearing phase (i.e. eggs didn’t hatch) or those with less than 51 data points in their home range polygons (see below). GPS locations where uploaded to ArcGIS 10.1 software and to movebank.org^46^ for further processing and archiving. The movement data used in this study are archived at movebank.org, study name “Movements of long-legged buzzards and short-toed eagles”, and are available through the Movebank Data Repository^47^.

All bird handling work were conducted according to relevant national and international guidelines, and were approved by the Israel Nature and Parks Authority (research permit *#*2011/38319).

### Large-scale foraging area

Foraging area for each tagged adult was defined by a 95% Kernel Density Estimator (KDE^28^) during each breeding season. We used the utilization distribution (UD) approach^28^, with a constant smoothing factor of 500 m. To assess the sensitivity of our results to the smoothing factor, we repeated the analysis using two additional smoothing factors, 250 m and 1000 m. We pre-processed the data in ArcGIS (ESRI. 10.1) and calculated the UD in R using the adehabitat package^48^. A foraging data point was defined as a point in which the ground speed was less than 4 m/s (hereafter “standing point”; Supplementary Fig. S7). A point-density analysis was used to identify foraging areas with high density of standing points. Using this method, all “flights corridors”, from the nest to the foraging areas, were excluded from the foraging area analysis. Our observations demonstrated that most of the activity in a radius of <0.15 km and <1.5 km from nests of LLB and STE, respectively, were dedicated to social interactions and not foraging; thus, were not included in the foraging-area analysis. For each individual we calculated the average distance between its nest and all the data points in its foraging area polygon.

Quality control for our analysis was composed of the following steps: (1) Polygons that contains less than 51 data points were excluded. (2) Tagged individuals that didn’t achieve the chick-rearing phase (i.e. eggs didn’t hatch) were excluded. (3) An average foraging area between two consecutive years was made for tagged individuals whose tag has been working throughout two consecutive breeding seasons (e.g. solar tags) and had similar foraging area during these two years (Supplementary Fig. S8).

LLBs tend to start incubating roughly three weeks prior to STEs, and complete their nesting approximately six weeks before STEs. Therefore, we analyzed our data in two separate time frames: (a) the entire breeding season from early incubation until one month after fledging for each species; and (b) the overlapping period (shared) within the breeding season of both species, i.e., from STEs’ early incubation until the end of LLBs’ post fledging phase (i.e. ~March 25^th^ – July 15^th^).

Pairwise attraction-repulsion relationships between each pair of birds in the nesting site was conducted using the MoveMine^49^ software. MoveMine determines the relationship by comparing the expected meeting frequency, as predicted using permutation of the observed tracks, with the actual meeting frequency in a pair of tracks^50^, where meeting is defined using proximity in space and time. Cluster analyses were conducted on known foraging points for individuals breeding in close proximity to one another. The *k*-means method of clustering, from the software MATLAB 2015a using the “kmeans” function, was utilized to classify foraging locations to spatially coherent groupings, assuming 5 clusters and an Euclidian distance function.

### Small-scale foraging habitat type

After determining the foraging areas, we identified and compared the foraging habitat types of the two raptor species within their foraging areas. We used the most current Israeli land cover GIS layer (KKL-JNF- Land cover survey of Israel, dataset 5, 2012). This dataset contains 15 categories of habitats within the study area; the four most common were: (1) Mediterranean rocky area, (2) Mediterranean Garrigue and Batha (hereafter low natural vegetation), (3) cultivated fields (e.g. all types of agricultural field crops: wheat, lucerne, potato etc.), and (4) uncultivated land (e.g. low vegetation leafy fields across the foothills valleys, sparse vegetation near dirt roads, exposed areas, etc.). The other 11 categories included: constructed land, quarries, breached areas, sandy areas, chaparral and forests 2–6 m height (dense, medium and sparse densities) and chaparral and forests 6–12 m height (dense, medium and sparse densities). The proportions of land cover of each of the 15 habitat types over the entire study area were determined using GIS. We identified the respective land cover type underneath each foraging standing point using GIS operations. This yielded a statistical distribution of habitat types in the foraging area, which is less prone to bias than a simple overlay of the entire KDE polygon on the land cover map.

### Timing-of-foraging analysis

We quantified and compared the foraging time for the two raptor species within their foraging areas. We monitored the time of foraging activity of each tagged individual of both species based on the timing of the position fix within their foraging area. We used only tagged individuals that achieved the chick-rearing phase and wore a solar tag, as their sampling frequency was sufficiently high to evaluate the timing of foraging in a consistent manner. Thus, timing of foraging was calculated for two and six individuals of STE and LLB, respectively. For each of these individuals we calculated the proportion of fixes during each daytime hour between 07:00–19:00.

### Diet analysis

All food remains and pellets were collected from each nest twice a year during three breeding seasons (2011–2013). In total we collected food remains from 182 nests of STE and 100 of LLB. The most widely used method for investigating raptors’ diet includes the analysis of both pellets and prey remains left at the nest^51^. We used a combination of these two methods to achieve a complete and reliable picture of the entire breeding diet^52^. All food remains and pellets were identified to the lowest possible taxonomic level. To prevent bias, we underestimated prey items as we considered all remains of one taxon that were found in one pellet as a single prey item^52^. Groups of feathers, fur or scales were recorded as a single prey item for each pellet or prey remain.

In total, 2,861 prey items were identified and categorized into 53 taxa, most of them to the species level (Supplementary Table S5). The identification of the reptile class was carried out by making a comprehensive scale index for most reptiles found in the Judean Foothills. By using this index, we were able to identify a species using a single dorsal scale. Mammals were identified by their bones, teeth, fur and nails while birds were identified by their feathers, bones, claws and legs. Arthropods were identified by their cuticles or the morphology of specific body parts.

In order to prevent bias we excluded all nests with samples which contained fewer than eight prey items during a breeding season. We have found that the two raptor species are monogamous and that pairs return to the same territories each year (Supplementary Fig. S8). To avoid pseudo-replication of nests among years we calculated the average number of each prey taxon per year for each nest. Consequently, all diet analyses were based on the average number of prey taxon of 32 and 59 nests for LLB and STE, respectively. For further calculations and statistical analyses we used either the frequency or the proportion of each prey taxa.

### Data analyses

We calculated foraging area overlaps within and between individuals of the two species based on the Utilization Distribution Overlap Index (UDOI)^29^ using R Package ‘adehabitat’^48^. For the comparison of the (1) habitat type within foraging area, (2) foraging timing, and (3) overall diet compositions of the two raptor species, data on (1) the proportions of habitat type used, (2) foraging time activity, and (3) prey taxa proportions, respectively were subjected to classification and ordination (PRIMER v6.2; www.primer-e.com)^53^. Analysis of similarity (ANOSIM)^54^, with the Global-R statistic was employed to test the null hypothesis of no difference between habitat type within foraging area, foraging timing and overall diet compositions of the two raptor species. We evaluated the interspecific niche overlap between the two species, and the intraspecific overlap among individuals within each raptor species with regard to habitat type use within their foraging area, foraging time and diet by using Pianka’s index^55^. To evaluate whether the pattern of the observed Pianka’s niche overlap diverged significantly from a random distribution (absence of overlap) we tested significance of the overlap by comparing the observed frequency of habitat type use, foraging timing and dietary prey taxa using randomization algorithms (RA3) by the ECOSIM 7 software^56^. We used rarefaction analysis to estimate the predicted total prey taxa richness in the diet of each of the two raptor species based on the frequency of prey taxa^57^. The dietary-niche breadth of each of the two raptor species was calculated by Levin’s index (*B*_*i*_). For full description of the above data analyses methodologies see Supplementary Methods.

### Data availability

The movement data used in this study are available on Movebank (movebank.org, study name “Movements of long-legged buzzards and short-toed eagles”) and are published in the Movebank Data Repository^47^.

## Additional Information

**How to cite this article**: Friedemann, G. *et al*. Multidimensional differentiation in foraging resource use during breeding of two sympatric top predators. *Sci. Rep.* **6**, 35031; doi: 10.1038/srep35031 (2016).

## Supplementary Material

Supplementary Information

## Figures and Tables

**Figure 1:**
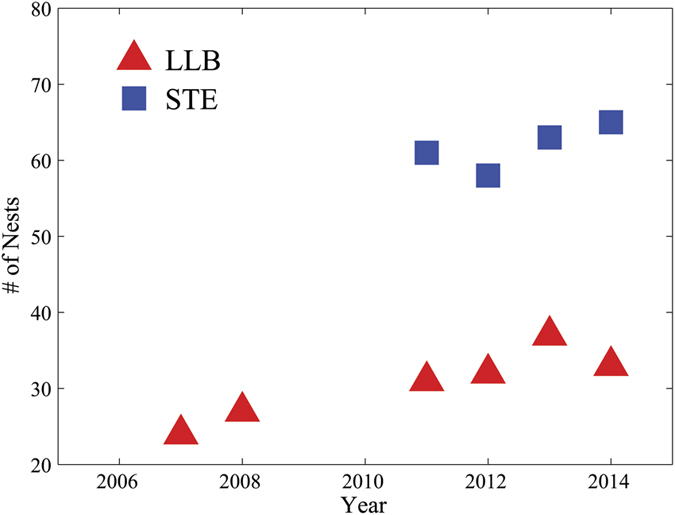
Number of nests located in the breeding area (215 km^2^) in the Judean Foothills, Israel. Note that long-legged buzzards (LLBs) and short-toed eagles (STEs) were not sampled during 2009–2010 and 2007–2010, respectively.

**Table 1:** Foraging area size and distance from their related nests, obtained by KDE (95%) analysis.

Species (n)	Distance of foraging area from nest (km) (mean ± SE)	Foraging area (km^2^) (mean ± SE)						
		Smoothing factor (h)	250 m		500 m		1000 m	
		Breeding season*	Whole	Shared	Whole	Shared	Whole	Shared
STE (8)	13.03 ± 2.20		59.1 ± 20.4	36.9 ± 14.7	95.4 ± 15.3	67.3 ± 13.5	152.1 ± 15.4	125.1 ± 13.4
LLB (9)	2.35 ± 0.62		15.0 ± 1.7	15.0 ± 1.7	22.7 ± 2.9	22.7 ± 2.9	43.5 ± 6.0	43.5 ± 6.0
Mann-Whitney U-Test	Z = 3.4 P = 0.001		3.08 <0.01	1.85 < 0.06	3.46 <0.01	2.70 <0.01	3.18 <0.01	3.23 <0.01

^*^= Whole = Entire breeding season for each species. Shared = From STEs’ early incubation till the end of LLBs’ post fledging phase.

**Table 2:** Multidimensional differentiation between long-legged buzzards (LLBs) and short-toed eagles (STEs).

Niche dimension	Long-legged buzzard (LLB)	Short-toed eagle (STE)
Geographic foraging area	Forage relatively close to their nests	Forage far from their nest
Foraging-habitat type	Forage at low natural vegetation, avoiding cultivated fields	Forage at cultivated fields, avoiding low natural vegetation
Diurnal dynamics of foraging	Uniformly active during daytime	Activity peaks in the early afternoon
Diet	Opportunistically diverse diet and consumed significantly higher percentages of lizards	Consumed significantly higher percentages of snakes in a more specialized manner
