# Supplementary material for: Multidimensional differentiation in foraging resource use during breeding of two sympatric top predators
Source: Sci Rep. 2016 Oct 11;6:35031. doi: 10.1038/srep35031 (PMC5057159; doi:10.1038/srep35031)
Supplement: Supplementary Information [file srep35031-s1.pdf]

Multidimensional differentiation in foraging resource use during breeding of two sympatric top predators

Guilad Friedemann[1,*], Yossi Leshem[1], Lior Kerem[1], Boaz Shacham[2], Avi Bar-Massada[3], Krystaal M McClain[4], Gil Bohrer[4], Ido Izhaki[5]

[1]*Department of Zoology, George S. Wise Faculty of Life Sciences, Tel Aviv University, Israel.*

[2]*National Natural History Collections, The Hebrew University of Jerusalem, Israel.*

[3]*Department of Biology and Environment, University of Haifa at Oranim, Kiryat Tivon, Israel.*

[4]*Department of Civil, Environmental and Geodetic Engineering, The Ohio State University, Columbus, OH, USA.* [5]*Department of Evolutionary and Environmental Biology, Faculty of Natural Sciences, University of Haifa, Israel.*

*Gilad.fridman@gmail.com

Supplementary Table S1. Spatial cluster analysis indicate that individuals' foraging locations clustered by species. Species values range from 0 to 1. The location of the foraging clusters (A-E) and foraging locations within them is shown in Fig 4a. Each cluster is composed of many foraging locations by several individuals. A single individual can be found foraging in multiple clusters; The number of individuals that foraged within the cluster is listed under “# Inds”. The number of individuals that foraged exclusively within that cluster is listed under “Unique Inds”. “Fraction of STE” shows the fraction of foraging locations by STE, out of the total foraging locations within the cluster, with 1, for example, indicate an exclusively STE cluster and 0 indicates an exclusive LLB cluster.

Foraging Cluster	# Inds	Unique Inds	Fraction of STE
**A**	4	2	0.001
**B**	6	1	0.748
**C**	5	1	1.000
**D**	8	4	0.057
**E**	3	0	0.977

Supplementary Table S2. Results of two types of SIMPER (similarity of percentages) analyses. (a) Similarity of habitat type used by each raptor species within their foraging areas and (b) dissimilarity between the two raptor species. Analyses show habitat types contributing most to similarity and dissimilarity in paired comparisons ranked by decreasing power (%). Mean abundance - the average abundance of each habitat type in bird's niche. Mean similarity/dissimilarity - the average contribution of each habitat type to the average similarity (analyses type a above) or dissimilarity (analysis type b above). The ratio dissimilarity/SD - dissimilarity/standard deviation, contribution (%) - mean contribution of each habitat type on the similarity or dissimilarity (analyses a and b above, respectively). Cutting point=80%.

	Similarity within each raptor species					
Land cover type	Average abundance	Average similarity	Contribution (%)	Cumulative contribution (%)		
	**LLB** Average similarity: 59.65					
Low natural vegetation	5.72	18.07	30.29	30.29		
Rocky area	4.32	14.79	24.79	55.08		
Uncultivated land	2.83	9.87	16.54	71.62		
Cultivated fields	2.7	5.7	9.56	81.17		
	**STE** Average similarity: 63.86					
Cultivated fields	7.55	31.98	50.08	50.08		
Rocky area	2.95	8.09	12.67	62.75		
Low natural vegetation	2.28	6.86	10.74	73.49		
Constructed area	2.08	5.73	8.97	82.46		
	Average dissimilarity between the two species = 49.72					
	Average abundance LLB	Average abundance STE	Average dissimilarity	Dissimilarity /SD	Contribution (%)	Cumulative contribution (%)
Cultivated fields	2.7	7.55	12.08	1.72	24.3	24.3
Low natural vegetation	5.72	2.28	9.56	1.67	19.24	43.54
Rocky area	4.32	2.95	5.88	1.01	11.82	55.36
Uncultivated land	2.83	1.31	4.66	1.63	9.37	64.74
Orchards	1.98	2.04	3.67	1.37	7.38	72.11
Constructed area	1.41	2.08	3.59	1.39	7.21	79.33
Mature forest	0.74	0.95	2.7	1.01	5.43	84.75

Supplementary Table S3. Probability test of RA3 null model between the mean observed and expected foraging habitat overlap, foraging time overlap and dietary niche overlap (Pianka's index, O_{jk}) between the two raptor species and among individuals within each species. The analysis for dietary niche overlap was calculated for each of three breeding seasons and all years combined. For foraging time and diet, the analysis assumes that all categories were equally available to both consumers. For the foraging habitats, we had independent estimates of availability and analyzed electivity. ns= not significant.

		RA3 Null Model	
	Mean Observed niche overlap (O_{jk})	Mean estimate (variance)	Pobs>Pexp<br><br>Pobs<Pexp
**Foraging habitat**			
Between species	0.24	0.39 (0.03)	ns
Within species			
LLB	0.39	0.17 (0.001)	0.001
STE	0.38	0.23 (0.001)	0.003
**Foraging time**			
Between species	0.86	0.86 (0.001)	ns
Within species			
LLB	0.98	0.97 (0.001)	0.02
STE	0.96	0.72 (0.006)	0.001
**Food items**			
Between species			
2011	0.61	0.18(0.014)	0.004
2012	0.33	0.16 (0.014)	ns
2013	0.43	0.16 (0.016)	0.04
All years	0.48	0.15 (0.011)	0.01
Within species LLB			
2011	0.87	0.18 (0.013)	0.001
2012	0.94	0.20 (0.012)	0.001
2013	0.85	0.18 (0.014)	0.001
Within species STE			
2011	0.94	0.16 (0.015)	0.001
2012	0.94	0.14 (0.014)	0.001
2013	0.91	0.14 (0.012)	0.001

Supplementary Table S4. Results of two types of SIMPER (similarity of percentages) analyses. (a) Similarity of foraging time activity within each raptor species and (b) dissimilarity between the two raptor species. For details see Supplementary Table S2.

	Similarity within each raptor species					
Foraging active hours	Average abundance	Average similarity	Contribution (%)	Cumulative contribution (%)		
	**LLB** Average similarity: 95.91					
**16-17h**	0.31	8.59	8.96	8.96		
**17-18h**	0.3	8.46	8.82	17.78		
**14-15h**	0.3	8.38	8.74	26.52		
**15-16h**	0.3	8.33	8.68	35.2		
**13-14h**	0.3	8.26	8.61	43.81		
**18-19h**	0.3	8.16	8.51	52.32		
**9-10h**	0.28	7.85	8.18	60.5		
**12-13h**	0.28	7.72	8.05	68.56		
**10-11h**	0.27	7.66	7.98	76.54		
**8-9h**	0.28	7.64	7.96	84.5		
**7-8h**	0.27	7.49	7.8	92.3		
	**STE** Average similarity: 90.21					
**12-13h**	0.38	11.7	12.97	12.97		
**13-14h**	0.37	11.45	12.7	25.67		
**11-12h**	0.37	11.26	12.49	38.16		
**14-15h**	0.36	10.72	11.88	50.04		
**10-11h**	0.33	9.71	10.76	60.8		
**15-16h**	0.33	9.15	10.15	70.95		
**16-17h**	0.28	7.48	8.29	79.24		
**9-10h**	0.26	7.07	7.84	87.07		
**17-18h**	0.18	5.5	6.09	93.17		
	Average dissimilarity between the two species = 17.13					
	Average abundance LLB	Average abundance STE	Average dissimilarity	Dissimilarity/ SD	Contribution (%)	Cumulative contribution (%)
**18-19h**	0.3	0.08	3.3	6.15	19.26	19.26
**7-8h**	0.27	0.11	2.53	2.23	14.75	34.01
**17-18h**	0.3	0.18	1.84	9.81	10.76	44.77
**8-9h**	0.28	0.16	1.81	1.9	10.56	55.33
**11-12h**	0.27	0.37	1.44	3.74	8.41	63.74
**12-13h**	0.28	0.38	1.43	4.46	8.36	72.11
**13-14h**	0.3	0.37	1.15	4.22	6.69	78.79
**10-11h**	0.27	0.33	0.91	2.3	5.29	84.08
**14-15h**	0.3	0.36	0.91	2.05	5.29	89.37
**16-17h**	0.31	0.28	0.68	1.8	3.96	93.33

Supplementary Table S5. Prey consumed by 32 LLB and 59 STE pairs, determined by analysis of pellets and prey remains in nests, during 2011-2013 breeding seasons. Results are expressed as the mean number (Mean ± SD) of prey taxa in each nests, and by its proportion (%). (All scientific and common names were stated according to most updated database http://www.reptile-database.org/).

**Prey**	**LLB nests (n = 32)**		**STE nests (n = 59)**	
	Mean ± SD	%	Mean ± SD	%
**Vertebrates**				
**Reptiles**				
**Colubridae**				
Large whip snake *Dolichophis jugularis*	1.09 ± 1.39	4.47	3.84 ± 2.35	22.86
Red whip snake *Platyceps collaris*	0.27 ± 0.55	1.09	1.29 ± 1.08	7.32
Montpellier snake *Malpolon insignitus*	0.28 ± 0.54	1.15	0.67 ± 0.98	4.01
Roth's dwarf racer *Eirenis rothii*	0	0	0.17 ± 0.13	0.1
Schokari sand racer *Psammophis schokari*	0.01 ± 0.06	0.04	0	0
European cat snake *Telescopus fallax*	0	0	0.02 ± 0.13	0.1
Unidentified little Colubridae	0.33 ± 0.47	1.37	0.36 ± 0.68	2.14
Unidentified large Colubridae	0.61 ± 0.98	2.5	2.33 ± 2.07	13.89
**Viperidae**				
Palestine viper *Daboia palaestinae*	0.51 ± 0.81	2.1	0.67 ± 0.94	3.97
**Boidae**				
Javelin sand boa *Eryx jaculus*	0	0	0.17 ± 0.13	0.1
**Anguidae**				
European glass lizard *Pseudopus apodus*	0.06 ± 0.25	0.26	0.03 ± 0.14	0.15
**Scincidae**				
Schneider's skink *Eumeces schneideri*	3.97 ± 4.43	16.32	0.42 ± 0.64	2.49
Bridled skink *Trachylepis vittata*	0.09 ± 0.32	0.38	0.16 ± 0.39	0.98
**Lacertidae**				
Lacerta laevis *Lacerta laevis*	0.05 ± 0.19	0.19	0	0
Unidentified Lacertidae	0.45 ± 1.77	1.84	0.32 ± 0.48	1.9
**Agamidae**				
Roughtail rock agama *Stellagama stellio*	3.55 ± 2.3	14.61	1.21 ± 1.35	7.21
**Chamaeleonidae**				
Mediterranean chameleon *Chamaeleo chamaeleon*	0.07 ± 0.22	0.3	0.47 ± 1.1	2.84
**Testudinidae**				
Mediterranean spur-thighed tortoise *Testudo graeca*	0.14 ± 0.4	0.58	0.24 ± 0.52	1.45
**Mammals**				
**Microtidae**				
Günther's vole *Microtus guentheri*	0.64 ± .018	2.63	0.25 ± 0.61	1.48
**Gerbillidae**				
Tristram's jird *Meriones tristrami*	0.33 ± 0.78	1.34	0.39 ± 0.63	2.27
**Muridae**				
Black rat *Rattus rattus*	0.04 ± 0.23	0.17	0.04 ± 0.2	0.25
House mouse *Mus Musculus*	0	0	0.02 ± 0.14	0.15
**Spalacidae**				
Palestine mole rat *Spalax ehrenbergi*	0.25 ± 0.51	1.01	0.08 ± 0.26	0.49
Unidentified Rodentia	2.65 ± 4.54	10.87	3.05 ± 3.45	18.19

**Leporidae**				
Cape hare *Lepus capensis*	0.25 ± 0.53	1.03	0	0
**Erinaceidae**				
East european hedgehog *Erinaceus concolor*	0.25 ± 0.51	1.01	0.06 ± 0.23	0.39
**Mustelidae**				
Marbled polecat *Vormela peregusna*	0.03 ± 0.18	0.12	0	0
**Procaviidae**				
Syrian rock hyrax *Procavia capensis syriaca*	0.03 ± 0.18	0.12	0	0
**Felidae**				
Wild cat *Felis silvestris*	0.01 ± 0.05	0.04	0.05 ± 0.04	0.03
**Birds**				
**Columbidae**				
Feral pigeon *Columba livia domestica*	2.61 ± 4.75	10.74	0.02 ± 1.45	0.15
**Phasianidae**				
Chukar *Alectoris chukar*	0.83 ± 0.81	3.42	0	0
**Corvidae**				
Western jackdaw *Corvus monedula*	0.26 ± 0.58	1.07	0	0
Eurasian jay *Garrulus glandarius*	0.43 ± 0.63	1.8	0	0
**Falconidae**				
Common kestrel *Falco tinnunculus*	0.41 ± 0.78	1.71	0	0
**Accipitridae**				
Eurasian sparrowhawk *Accipiter nisus*	0.01 ± 0.08	0.06	0	0
Short-toed eagle *Circaetus gallicus*	0.01 ± 0.08	0.06	0	0
**Strigidae**				
Scops owl *Otus scops*	0.01 ± 0.08	0.06	0	0
Little owl *Athene noctua*	0.03 ± 0.18	0.12	0	0
**Ardeidae**				
Cattle egret *Bubulcus ibis*	0.14 ± 0.32	0.59	0	0
**Phoeniculidae**				
Hoopoe *Upupa epops*	0.04 ± 0.18	0.17	0	0
**Alcedinidae**				
White-throated kingfisher *Halcyon smyrnensis*	0.03 ± 0.18	0.13	0	0
Unidentified Bird	2.98 ± 2.5	12.23	0.03 ± 0.18	0.2
**Invertebrates**				
**Tettigonioidea**				
White-forehead bush-cricket *Decticus albifrons*	0.15 ± 0.55	0.64	0.1 ± 0.29	0.64
Carnivore grasshopper *Saga ephippigera*	0.03 ± 0.17	0.13	0.02 ± 0.11	0.15
**Buprestidae**				
Middle eastern jewel beetle *Steraspis squamosa*	0.01 ± 0.09	0.06	0.33 ± 0.69	1.95
**Scarabaeinae**				
Spanish copris *Copris hispanus*	0.03 ± 0.12	0.13	0.01 ± 0.13	0.1
**Tenebrionidae**				
Darkling beetle *Adesimia abbreviata*	0.03 ± 0.17	0.13	0	0
**Spirostreptidae**				
Archispirostreptus syriacus	0.03 ± 0.17	0.13	0	0
**Scorpiones**				
Dark large clawed scorpion *Scorpio fuscus*	0.06 ± 0.24	0.25	0.12 ± 0.35	0.73
Israeli gold scorpion *Scorpio palmatus*	0.1 ± 0.05	0.04	0.17 ± 0.13	0.1
Unidentifed Scorpiones	0.04 ± 0.19	0.19	0.04 ± 0.19	0.25
Unidentifed Araneae	0.01 ± 0.08	0.06	0	0
Unidentifed Invertebrates	0.12 ± 0.3	0.5	0.09 ± 0.32	0.57

Supplementary Table S6. Results of two types of SIMPER (similarity of percentages) analyses. (a) Similarity of dietary prey items within each raptor species and (b) dissimilarity between the two raptor species. For details see Supplementary Table S2.

	Similarity within each raptor species					
Prey item	Mean abundance	Mean similarity	Contribution (%)	Cumulative contribution (%)		
	**LLB** Average similarity: 48.44					
Roughtail rock agama	0.38	9.77	20.18	20.18		
Schneider's skink	0.35	8.70	17.97	38.15		
Unidentified bird	0.32	7.54	15.57	53.72		
Feral pigeon	0.29	6.19	12.78	66.50		
Unidentified Rodentia	0.24	4.69	9.69	76.19		
Chukar	0.15	2.58	5.33	81.52		
	**STE** Average similarity: 51.59					
Large whip snake	0.48	15.69	30.42	30.42		
Unidentified large Colubridae	0.34	8.47	16.42	46.83		
Unidentified Rodentia	0.31	6.72	13.02	59.85		
Red whip snake	0.24	6.04	11.71	71.56		
Roughtail rock agama	0.22	4.60	8.92	80.48		
	Average dissimilarity between the two species = 70.94					
	Mean abundance LLB	Mean abundance STE	Mean dissimilarity	Dissimilarity/ SD	Contribution (%)	Cumulative contribution (%)
Large whip snake	0.15	0.48	5.97	1.82	8.41	8.41
Unidentified bird	0.32	0.01	5.37	1.94	7.58	15.99
Feral pigeon	0.29	0.01	5.16	1.33	7.28	23.27
Unidentified Large Colubridae	0.10	0.34	5.16	1.29	7.27	30.54
Schneider's skink	0.35	0.09	4.99	1.66	7.04	37.57
Unidentified Rodentia	0.24	0.31	4.05	1.36	5.71	43.28
Roughtail rock agama	0.38	0.22	3.98	1.28	5.61	48.89
Red whip snake	0.05	0.24	3.81	1.52	5.38	54.26
Chukar	0.15	0.00	2.65	1.15	3.73	57.99
Palestine viper	0.09	0.14	2.54	1.07	3.58	61.57
Montpellier snake	0.05	0.14	2.51	0.98	3.53	65.11
Unidentified Lacertidae	0.05	0.09	1.84	0.86	2.59	67.70
Tristram's jird	0.06	0.08	1.77	0.86	2.49	70.19
Unidentified little Colubridae	0.07	0.08	1.76	0.97	2.48	72.68
Mediterranean chameleon	0.02	0.09	1.70	0.70	2.39	75.07
Eurasian jay	0.09	0.00	1.61	0.66	2.27	77.34
Günther's vole	0.06	0.05	1.49	0.65	2.10	79.44
Mediterranean spur - thighed tortoise	0.03	0.06	1.31	0.59	1.85	81.30

Supplementary Figure S7. Total movements during the breeding season of two STEs (#2186-a, c) and two LLBs (b, d), divided into "flying data points" (pale blue) and "standing data points" (red). These figures were generated by using GIS software - ArcGIS (ESRI. 10.1).

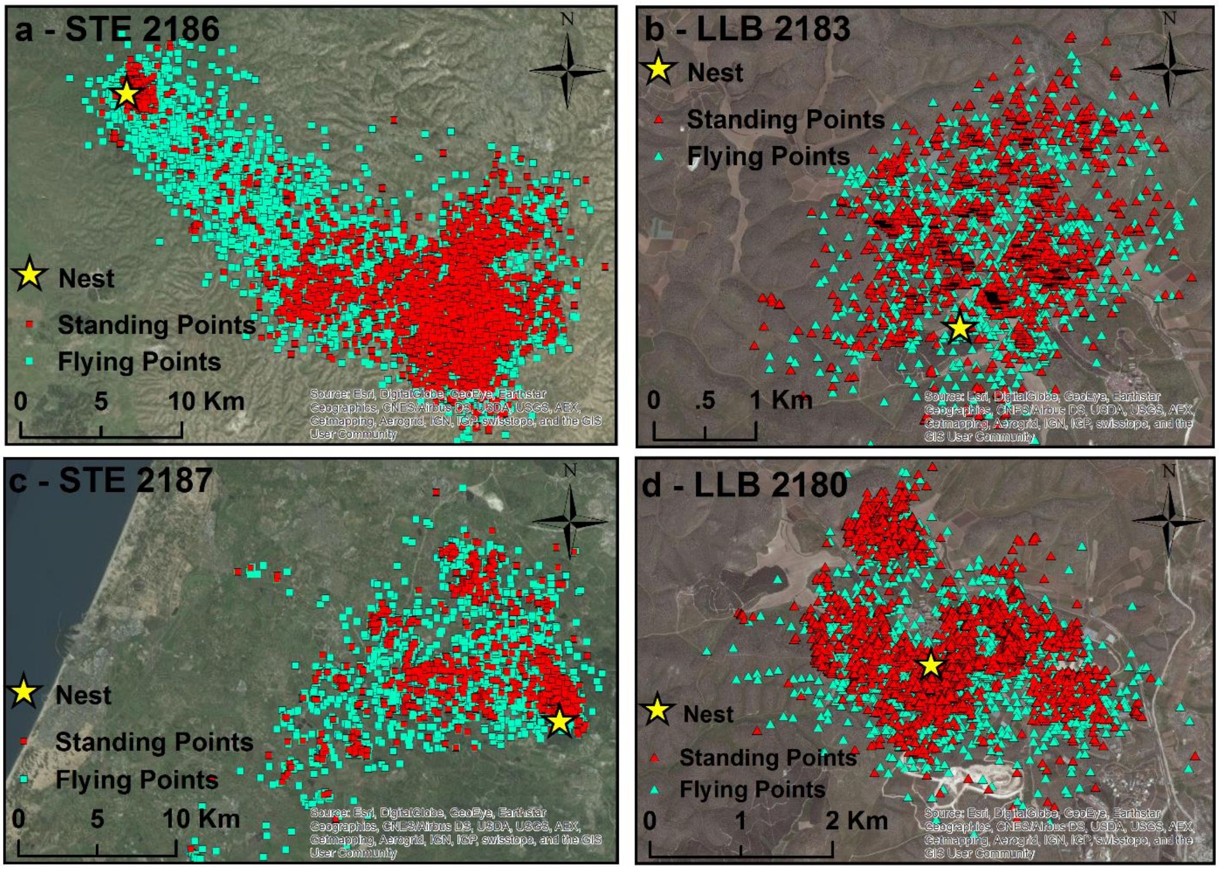

Supplementary Figure S8. Total movements during the breeding season of one STE (#2186) in 2012 (a) and 2013 (c), and one LLB (#2180) in 2012 (b) and in 2013 (d). Note that each year the two raptor species are using the same movement pattern and foraging area size and location. These figures were generated by using GIS software - ArcGIS (ESRI. 10.1).

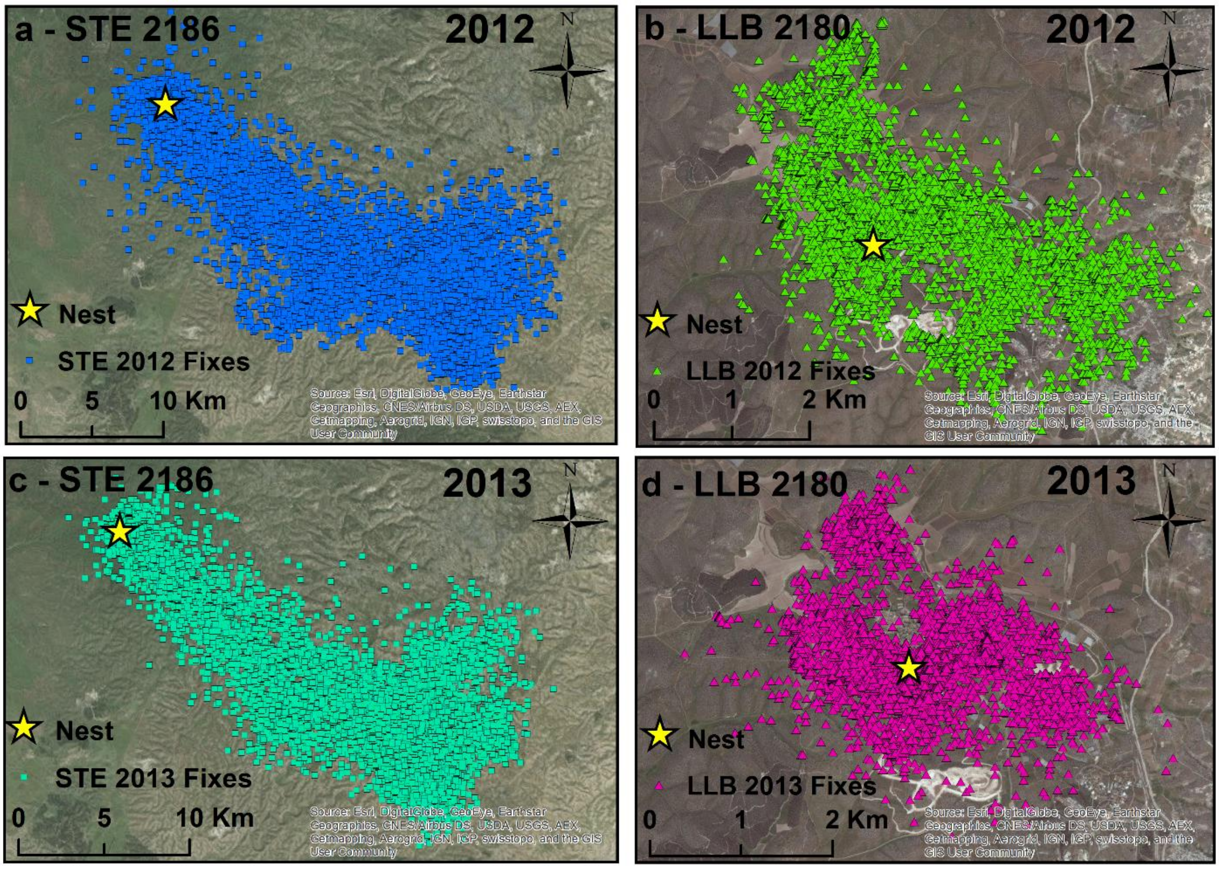

Supplementary Methods

Studied species

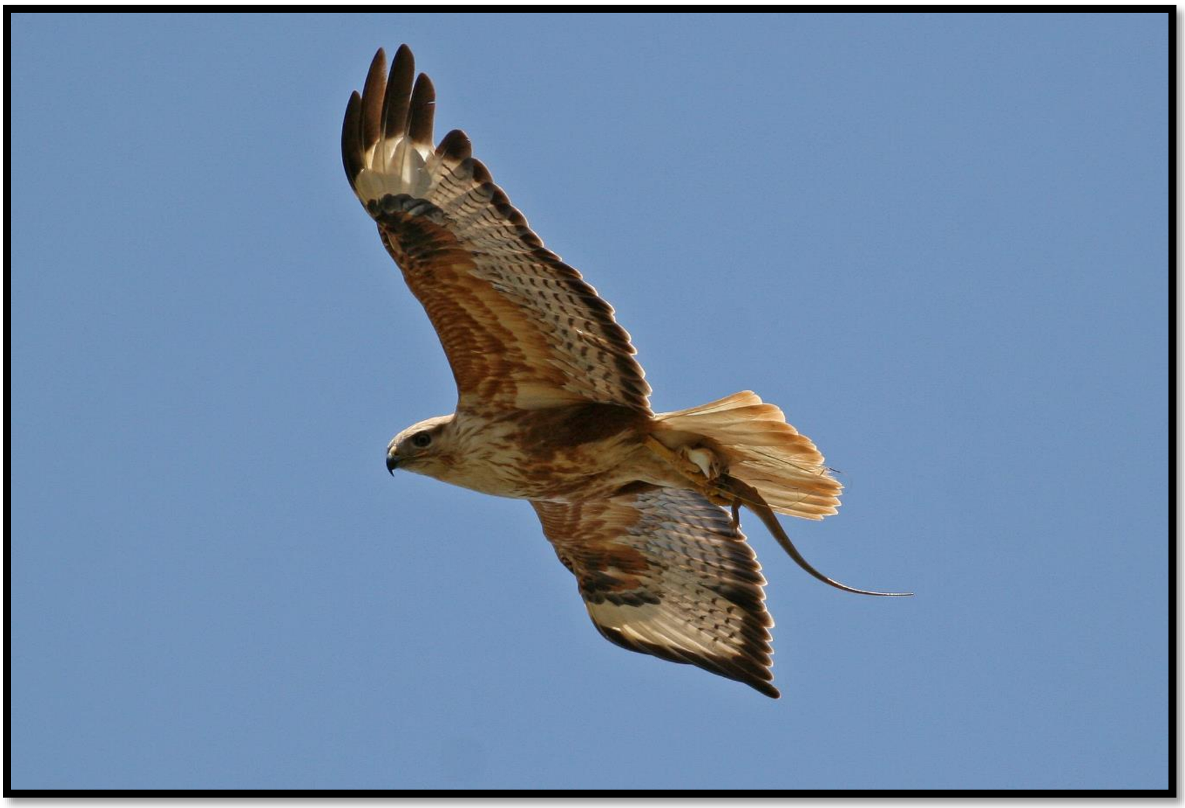

Long-legged buzzard (*Buteo rufinus*, LLB) is known as a large size (weight: 1,100g-1,600g; wing span: 115-170cm), shy and aggressive species and therefore it has been seldom studied. Most studies have focused on its nest location[1-4] and/or analyzed small samples of food remains from a relatively small number of nests[2, 5-7]. To date, most studies largely overlooked its foraging habitat preferences, foraging movements, and full diet. Until the 1980s at least 31 pairs of LLBs nested along the cliffs of the Judean Mountains. During the past 40 years, however, nesting in these areas has substantially decreased, with at least 45 pairs of LLBs now nesting in a new area - the Judean Foothills, but on trees rather than cliffs[8] as opposed to the published literature[1, 9, 10].

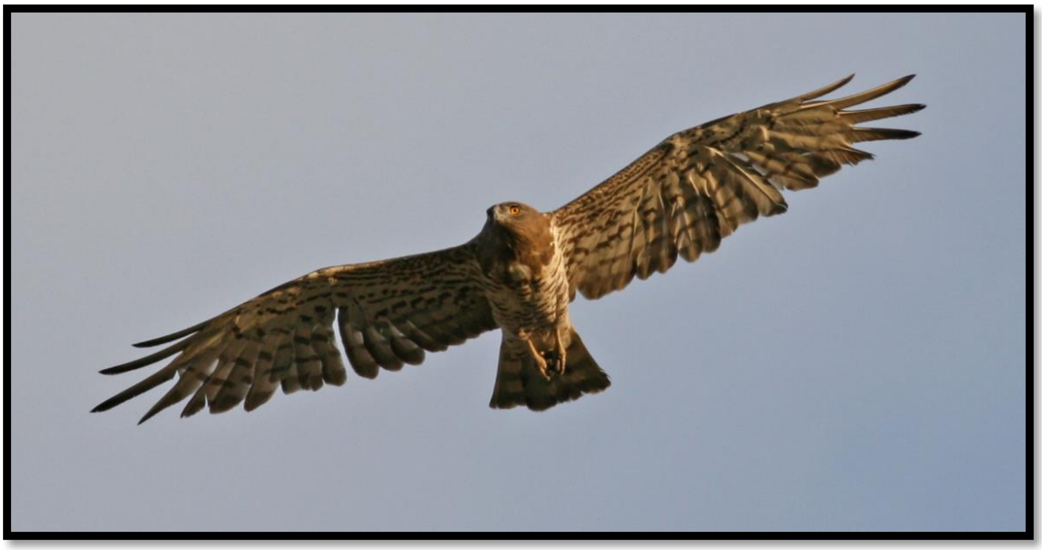

Short-toed eagle (*Circaetus gallicus*, STE) is a large-sized raptor (weight: 1,200g-2,300g; wing span: 165-185cm), which adopted the Judean Foothills as its main historical nesting area (~100 pairs). In a previous study[11] it was found that LLB diets comprise a significant amount of reptiles (40-50%), almost as similar as the diet of STEs, which comprises almost entirely reptiles[12-14].

Data analysis

We calculated foraging area overlaps within and between individuals of the two species based on the Utilization Distribution Overlap Index (UDOI)[15] using R Package 'adehabitat'[16]. The UDOI is denoted by:

$$(1) \qquad UDOI = A_{i,j} \int_x \int_y UDi(x,y) X \, UDj(x,y)$$

Where $A_{i,j}$ is the area of the intersection between the two foraging ranges and UD*i*(x,y) is the value of the utilization distribution of the bird *i* at the point (x,y). UDOI values range between 0 and 1, where an overlap value of 1 indicates complete overlap, whereas 0 indicates no overlap between adjacent UDs. UDOI seems to be the most appropriate technique for quantifying overlap in terms of shared space use, particularly for studies using kernel-based estimates of home range (e.g., kernels) and GPS telemetry[15].

For the comparison of the (1) habitat type within FA, (2) foraging timing and (3) overall diet compositions of the two raptor species, data on (1) the proportions of habitat type used, (2) foraging time activity, and (3) prey taxa proportions, respectively were subjected to classification and ordination (PRIMER v6.2; www.primer-e.com)[17]. The data were root transformed, and a similarity matrix was constructed using the Bray-Curtis similarity coefficient. The resulted resemblance matrix was employed to generate non-metric multi-dimensional scaling (MDS) ordination plots and group-averaged hierarchical clustering dendrograms. Low stress values (< 0.20) in the MDS analysis indicated that two-dimensional axes were sufficient to achieve reliable and robust separation of data[18]. Similarity contours were derived from the dendrogram and applied to the MDS ordination plots in PRIMER 6.2.

Analysis of similarity (ANOSIM)[19], with the Global-R statistic was employed to test the null hypothesis of no difference between habitat type within FA, foraging timing and overall diet compositions of the two raptor species. ANOSIM *R* is a non-parametric permutation procedure that uses the underlying Bray-Curtis resemblance matrix to rank similarities among a priori designations. ANOSIM calculates a global R statistic from the fraction of the difference between average rank similarity within groups and average rank similarity between groups over a function of the number of samples. R-statistic values range from 0 (no separation between the two groups) to 1 (complete separation between the two groups). Typically, if R is within 0.3 to zero, then the differences between groups are considered to be negligible[17]. Low R values can be significant if sample sizes are large. Next, we employed the similarity percentages (SIMPER) analysis to identify the habitat type, foraging hour and prey taxa contributing most to the separation between the two raptor species and to the similarity among individuals within each species[18].

We evaluated the interspecific niche overlap between the two raptor species and the intraspecific overlap among individuals within each raptor species with regard to habitat type use within their FA, foraging time and diet in by using Pianka's index:

(2) $$O_{jk} = \frac{\sum_{i}^{n} p_{ij} p_{ik}}{\sqrt{\sum_{i}^{n} p_{ij}^2 \sum_{i}^{n} p_{ik}^2}}$$

Where p_i is the frequency of occurrence of habitat type (i = 15 categories), foraging timing (i = 12 categories) or prey taxa (i = 53 categories) in species j and k[20]. The range of Pianka's index (O_{jk}) is between 0 (total separation) and 1 (total overlap). A basic assumption adopted here was that the different dietary resources were equally accessible to both raptor species, given that no data was collected on the availability of resources within the study area[21]. We also assumed that the time of foraging activity during the daytime was equally available for both species. However, habitat type use was compared to the actual availability of each habitat type within the study area.

In order to evaluate whether the pattern of the observed Pianka's niche overlap diverged significantly from a random distribution (absence of overlap) we tested for significance of the overlap by comparing the observed frequency of habitat type use, foraging timing and dietary prey taxa with values obtained by randomizing the original matrices (30,000 iterations) using a randomization algorithms (RA3) by the ECOSIM 7 software[22]. The RA3 algorithm has been shown to have desirable statistical properties for detecting nonrandom niche overlap patterns as it is robust to Type 1 and Type 2 errors[22]. The RA3 algorithm conserves niche breadth values (i.e., degree of specialization) but randomly reshuffles these values, including any zeros, among categories for each species[23]. Thus, we used RA3 to retain the amount of specialization for each species, but allow it to potentially use other resource categories[23]. Using this methodology we analyzed intraspecific and interspecific niche overlap between individuals of each raptor and between the two species, respectively, all years combined. We also analyzed diet overlap for each year separately. For foraging time and diet we assumed that all categories were equally available to both species. For the foraging habitats

we had independent estimates of availability and thus corrected the model by the electivity index[22].

We used rarefaction analysis to estimate the predicted total prey taxa richness in the diet of each of the two raptor species based on the frequency of prey taxa[24]. We calculated the Chao1 index, which is a nonparametric abundance-based richness estimator. This index is recommended as the best estimate for abundance-based richness when Chao's estimated CV for incidence distribution is > 0.5 as was found in our case. To remove the effect of sampling order, the sampling order was randomized 1,000 times without replacement and mean prey taxa richness estimate was computed for each sample accumulation level. Predicted (rarefaction) taxa accumulation curves were calculated using the software: EstimateS 7.52[24].

The dietary niche breadth of each of the two raptor species was calculated by Levin’s index (B_i):

$$(3) \qquad B_i = 1/\sum_{i=1}^{n} P_i^2$$

Where P_i is the relative occurrence of prey taxon i in a given species' diet and n is the number of prey taxa actually consumed by a given raptor species. In practice, this index measures the niche breadth based on Simpson's diversity index. This index generates values between 1 and n (when n resources are used equally).

A Mann-Whitney U Test was carried out in order to compare the foraging area size and the distance of the foraging area points from the nest between the two raptor species. One sample t–test was used to compare habitat type use of each species in comparison to habitat type availability in the whole study area. We used two independent sample t-tests for the comparison of the distance between nests of the two species and for the comparison of the total proportions of each prey category (reptiles, birds, mammals and arthropods) between the two raptor species. A repeated measures ANOVA analysis was used in order to compare between

the consumption of lizards (Sauria) versus snakes (Ophidia, including the European glass lizard, *Ophisaurus apodus*, due to its snake-shape structure), between the two raptor species.

References

1. Alivizatos, H., Goutner, V. & Karandinos, M. G. Reproduction and behaviour of the long-legged buzzard (*Buteo rufinus*) in north-eastern Greece. *Vogelwarte* **39**, 176-182 (1998).

2. Milchev, B. Breeding biology of the long-legged buzzard *Buteo rufinus* in SE Bulgaria, nesting also in quarries. *Avocetta* **33**, 25-32 (2009).

3. Baltag, E. S., Bolboaca, L. E. & Ion, C. Long-legged buzzard (Aves: *Buteo*) breeding population from Moldova region. *Eur. Sci. J.* **2**, 346-351 (2014).

4. Demerdzhiev, D., Dobrev, V. & Popgeorgiev, G. Effects of habitat change on territory occupancy, breeding density and breeding success of long-legged buzzard (*Buteo rufinus* Cretzschmar, 1927) in Besaparski Ridove special protection area (Natura 2000), Southern Bulgaria. *Acta. Zool. Bulgar.* **5**, 191-200 (2014).

5. Alivizatos, H. & Goutner, V. Feeding habits of the long-legged buzzard (*Buteo rufinus*) during breeding in northeastern Greece. *Isr. J. Zool.* **43**, 257-266 (1997).

6. Wu, Y. Q. Food habitats of the breeding long-legged buzzard (*Buteo rufinus*) in northwestern China. *Appl. Mech. Mater.* **71**, 2699-2702 (2011).

7. Bakaloudis, D. E. *et al*. Assessing bias in diet methods for the long-legged buzzard *Buteo rufinus*. *J. Arid Environ.* **77**, 59-65 (2012).

8. Friedemann, G., Yom-Tov, Y., Motro, U. & Leshem, Y. Shift in nesting ground of the long-legged buzzard (*Buteo rufinus*) in Judea, Israel - An effect of habitat change. *Biol. Conserv.* **144**, 402-406 (2011).

9. Cramp, S. & Simmons, K. E. L. *The Birds of the Western Palearctic*. (Oxford University Press, Oxford, 1980).

10. Shirihai, H. *The Birds of Israel*. (Academic Press, London, 1996).

11. Friedemann, G., Yom-Tov, Y., Motro, U. & Leshem, Y. The breeding biology of the Judean long legged buzzard (*Buteo rufinus*), Israel. (2009).

12. Vlachos, C. G. & Papageorgiou, N. K. Diet, breeding success, and nest-site selection of the short-toed eagle (*Circaetus gallicus*) in northeastern Greece. *J. Raptor Res*. **28**, 39-42 (1994).

13. Moreno-Rueda, G. & Pizarro, M. Snake species richness and shrubland correlate with the short-toed eagle (*Circaetus gallicus*) distribution in southeastern Spain. *Ann. Zool. Fenn.* **44**, 314-320 (2007).

14. Bakaloudis, D. E. & Vlachos, C. G. Feeding habits and provisioning rate of breeding short-toed eagles *Circaetus gallicus* in northeastern Greece. *J. Biol. Res-Thessalon*. **16**, 166-176 (2011).

15. Fieberg, J. & Kochanny, C. O. Quantifying home-range overlap: the importance of the utilization distribution. *J. Wildl. Manage.* **69**, 1346-1359 (2005).

16. Calenge, C. The package "adehabitat" for the R software: a tool for the analysis of space and habitat use by animals. *Ecol. Model.* **197**, 516-519 (2006).

17. Clarke, K. R. & Gorley, R. N. "Primer". User manual/tutorial. *PRIMER-E Ltd.*, Plymouth, UK. (2006).

18. Clarke, K. R. Nonparametric multivariate analyses of changes in community structure. *Aust. J. Ecol.* **18**, 117-143 (1993).

19. Quinn, G. P. & Keough, M. J. in *Experimental Design and Data Analysis for Biologists* xvii, 1-537 (Cambridge University Press, Cambridge, UK, 2002).

20. Pianka, E. R. The structure of lizard communities. *Annu. Rev. Ecol. Syst.* 53-74 (1973).

21. Fedatto Abelha, M. C., Goulart, E., Luiz Kashiwaqui, E. A. & da Silva, M. R. *Astyanax paranae Eigenmann*, 1914 (Characiformes: Characidae) in the Alagados Reservoir, Parana, Brazil: diet composition and variation. *Neotrop. Ichthyol.* **4**, 349-356 (2006).

22. Gotelli, N. J. & Entsminger, J. L. EcoSim: null models software for ecology. Acquired Intelligence Inc. & Kesey-Bear. (2006).

23. Gotelli, N. J. & Graves, G. R. in *Null Models in Ecology*. xvi+368p, (1996).

24. Colwell, R. K. EstimateS: Statistical estimation of species richness and shared species from samples, Version 7.52. User's guide and application published online. URL http://viceroy.eeb.uconn.edu/estimates. (2009).
